# Beyond the myths: Novel findings for old paradigms in the history of the smallpox vaccine

**DOI:** 10.1371/journal.ppat.1007082

**Published:** 2018-07-26

**Authors:** José Esparza, Andreas Nitsche, Clarissa R. Damaso

**Affiliations:** 1 Institute of Human Virology, University of Maryland School of Medicine, Baltimore, Maryland, United States of America; 2 Centre for Biological Threats and Special Pathogens 1 –Highly Pathogenic Viruses & German Consultant Laboratory for Poxviruses & WHO Collaborating Centre for Emerging Infections and Biological Threats, Robert Koch Institute, Berlin, Germany; 3 Laboratório de Biologia Molecular de Virus, Instituto de Biofísica Carlos Chagas Filho, Universidade Federal do Rio de Janeiro, Rio de Janeiro, Brazil; University of Florida, UNITED STATES

## Introduction

The discovery of smallpox vaccination by Edward Jenner in 1796 gave a new perspective to science in the struggle against this devastating disease. Smallpox has claimed hundreds of millions of lives over the centuries. However, it was only in 1980 that the World Health Organization finally declared smallpox eradicated after an intense worldwide vaccination campaign [1].

The intriguing history of the smallpox vaccine is replete with mythology that continues to fascinate researchers today. The main mystery concerns the true origin of the vaccine matter used by Jenner and subsequent early vaccinators [2, 3]. For the 20th century, we certainly know the answer: vaccinia virus. But what of previous centuries—was cowpox or horsepox the virus used? Or was it actually vaccinia virus that was being used, and if so, how did this come about? After all, vaccinia virus has never been reported to cause natural infections in animals, except for escapee vaccine strains in Brazil and India [4]. Next-Generation Sequencing (NGS) technologies applied to genomic studies of modern and old smallpox vaccines are helping to sort out these puzzles.

### Demystifying Jenner reveals an even more powerful figure

In 1796, the British country doctor Edward Jenner decided to test the hypothesis that previous contact with a disease of cattle known as cowpox would prevent the development of smallpox. The disease induced pustular lesions localized to the teats of dairy cattle and the hands of milkmaids, which resembled smallpox lesions. In one experiment, Jenner scarified the arm of an 8-year-old boy with the material of a cowpox lesion obtained from the hand of a milkmaid, and 6 weeks later, he scarified the arm of the boy again with smallpox-derived material. Because the boy did not develop smallpox, Jenner was convinced of the “preventive power” of cowpox. These and other findings were gathered in a manuscript published in 1798, and a couple of years later, the procedure was named vaccination (from Latin “vacca,” “cow”) [5]. As time passed, history continued to faithfully relay the information that the smallpox vaccine contains cowpox virus [6].

But what actually formed the basis for protection? Different from most current vaccines, the material used for smallpox vaccination was not an attenuated or inactivated form of the virus that caused the disease: variola virus. Instead, it was a related virus from the same family (Poxviridae) and genus (*Orthopoxvirus*). Today, we know that infection with one orthopoxvirus elicits cross-immunity against subsequent infection with another orthopoxvirus in humans [7]—something that Jenner could not have known since viruses did not begin to be identified until the end of the 19th century. Thus, Jenner was unaware of the true biological nature of the vaccine material, which was called indistinctly cowpox virus or vaccinia virus.

**Fig 1:**
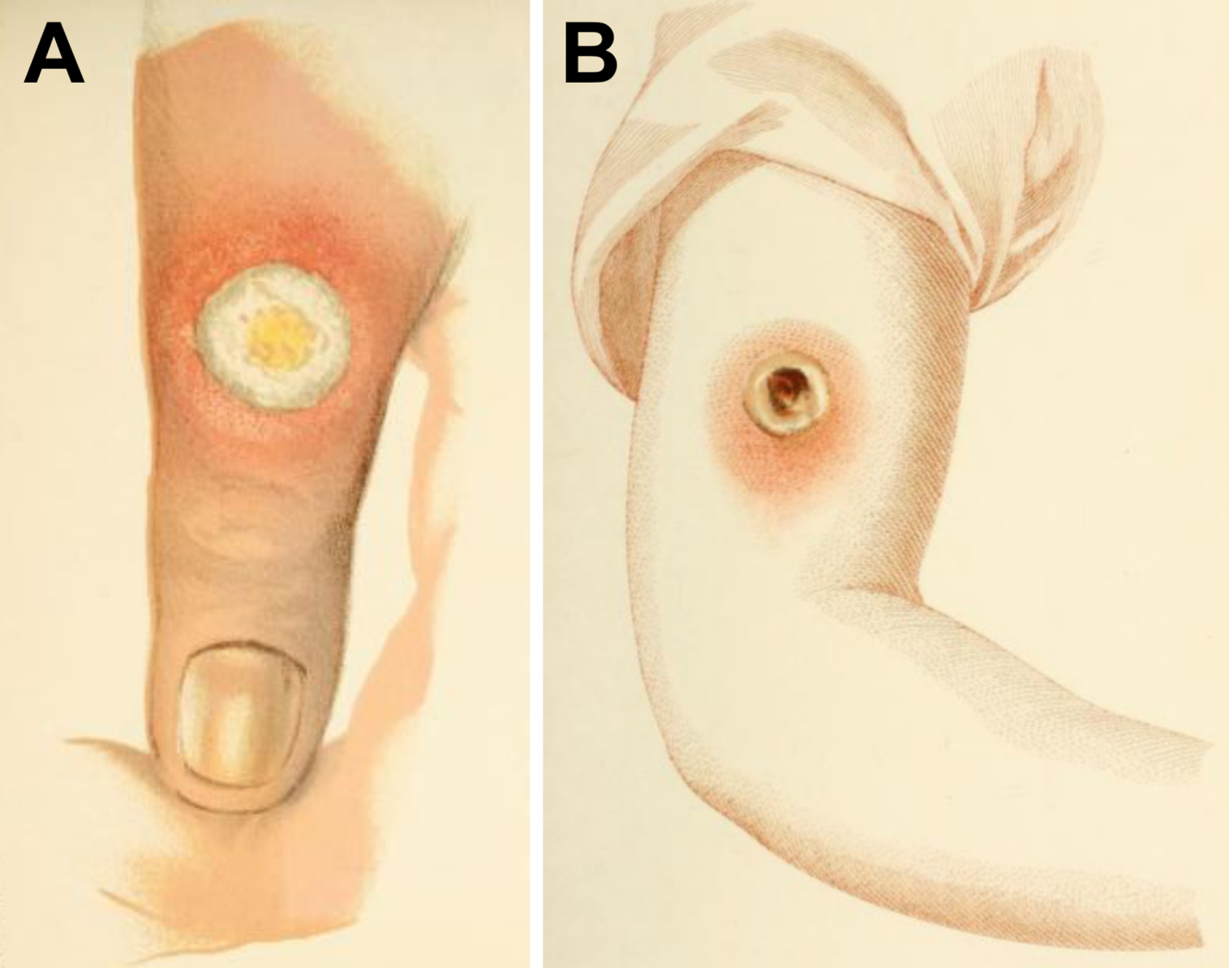
Cowpox and horsepox lesions. (A) Old illustration depicting a cowpox lesion on the finger of milker William Plowman 5 days after the onset of vesicle formation. December 2, 1887. (B) Old illustration depicting a horsepox lesion on the arm of 5-year-old John Baker, who was inoculated with the material taken from a horsepox lesion (equination) on the hand of a stableman by Edward Jenner. March 16, 1798. Reproduced for noncommercial research purposes from reference [8].

Many other experimental inoculations were reported by Jenner in the same manuscript, and in some cases, the vaccine material was obtained from lesions of horses affected by the so-called horsepox, a disease similar to cowpox. The horse lymph was scarified directly on a person’s arm or first inoculated in cows, and then the cow lymph was passed to a person (Fig 1). Actually, Jenner was convinced that the best protective results were obtained when the transmission chain horse–cow–human was followed [5, 8]. This procedure was referred to as equination, and Jenner, as well as other British and Italian physicians, used it successfully in the following years [2, 3]. But was that disease of horses caused by the orthopoxvirus we know today as horsepox virus? Again, this was impossible to know at the time.

Over the centuries, numerous myths have been associated with Jenner’s discovery in attempts to either deify him or criticize him [9]. However, stripped of all the myths, Jenner emerges as an even more powerful figure. Three major contributions clearly stand out as his legacy: (1) through careful observation and experimentation, he provided scientific evidence for the role of “cowpox” in the prevention of smallpox, even in the absence of the theoretical framework provided by the germ theory of disease, which was formulated some 80 years later; (2) he found that the vaccine material against smallpox could be serially transferred in humans (also called Jennerian or humanized vaccine), establishing a practical arm-to-arm system for spreading the vaccine worldwide; and (3) he championed vaccination as a public health intervention, predicting that vaccination would one day “annihilate” smallpox [10].

### Worldwide distribution of the smallpox vaccine

The Jennerian vaccine spread rapidly worldwide, and all European countries, the Americas, and Asia had the vaccine by 1813 [11]. As stocks were depleted, they were renewed by fresh pustular material from naturally infected cows and/or horses. Therefore, vaccine stocks changed massively over the following years [3].

After the mid-1800s, the propagation method for smallpox vaccine changed to sequential passage of lymph material in cows (animal vaccine). Italy and France played a distinguished role in this process, and vaccine production increased worldwide. Different vaccine strains produced in different establishments were exchanged globally and mixed empirically in attempts to generate vaccines with increased potency [3].

### The true nature of the smallpox vaccine: Cowpox, horsepox, or vaccinia virus?

A watershed in the history of the smallpox vaccine came in 1939 when British researcher Allan Downie, using immunological assays, made a clear distinction between the virus in the smallpox vaccine (vaccinia virus) and viruses that had been obtained from natural cases of cowpox [12]. This work raised important questions to researchers that we have not yet been able to answer: When and how did vaccinia virus start being used as the smallpox vaccine? Conversely, was cowpox virus or horsepox virus ever used? Why are all extant 20th century smallpox vaccines made with vaccinia virus and none with cowpox virus?

These questions are just part of a more complicated conundrum. Cowpox and vaccinia are different orthopoxviruses, while horsepox virus belongs to the vaccinia lineage. As orthopoxviruses, we expect that they all can elicit cross-protection against smallpox [7]. Cowpox and vaccinia viruses can infect cows, humans, and horses, generating similar pustular diseases that prove difficult to distinguish clinically [3]. As for horsepox, we expect the same since the virus belongs to the vaccinia lineage. It is known that the Mongolian strain can infect horses [13], and the Mulford strain—a horsepox-like virus—was reported to infect cows and humans in the early 20th century [14]. What is more, the natural hosts and reservoirs of cowpox virus are rodents, not cows [7, 15]. Similarly, rodents have been reported as reservoirs for feral strains of vaccinia virus in Brazil [16], and this is probably true for horsepox virus. Hence, cows, horses, and humans are all accidental hosts! To the best of our knowledge, today we can only conclusively state that Jenner vaccinated people with an orthopoxvirus.

### Modern sequencing technologies to the rescue

**Fig 2:**
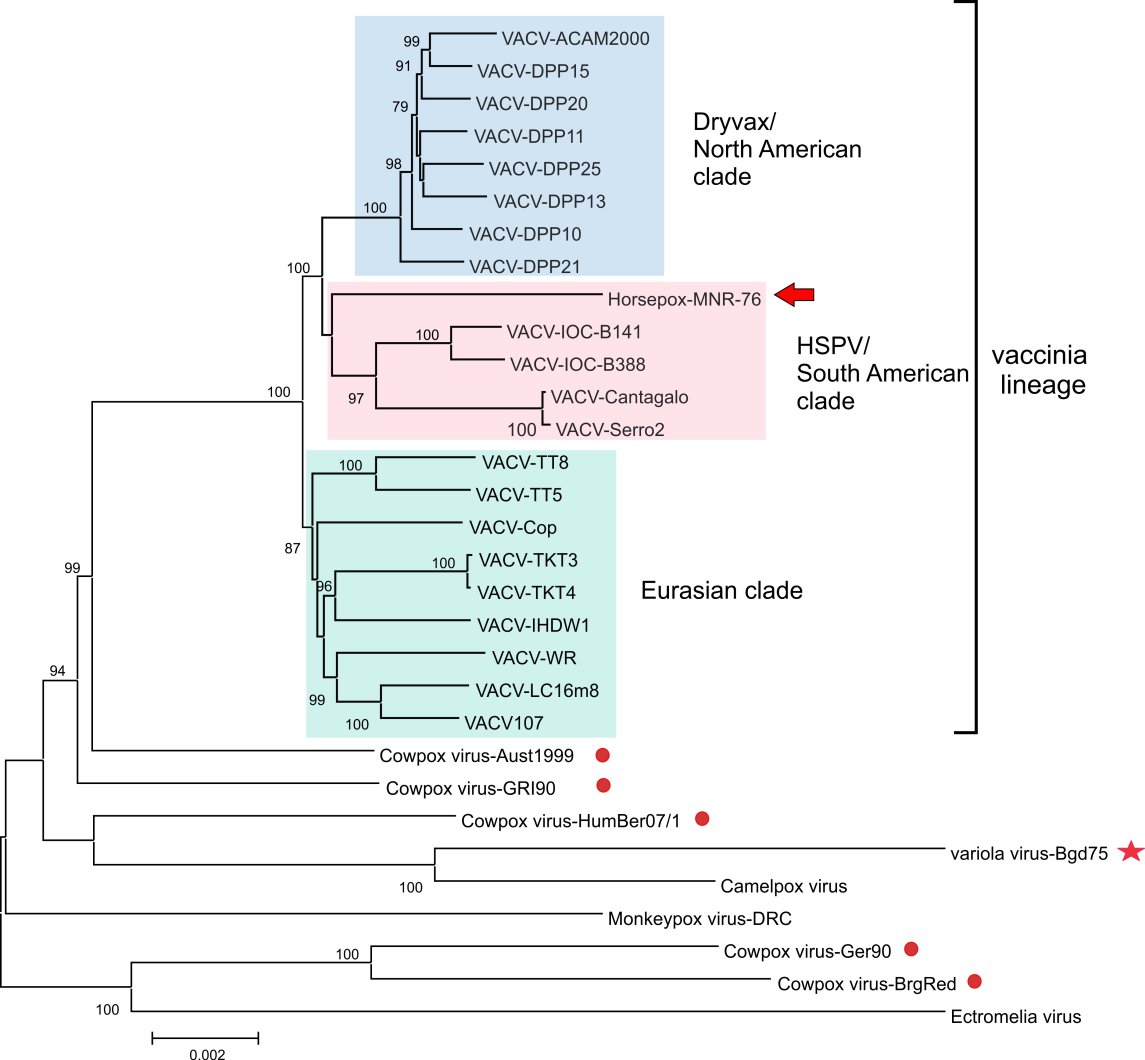
Phylogenetic tree of the vaccinia virus lineage. Phylogeny inference (a neighbor-joining tree with use of the Kimura 2-parameter substitution model and 1,000 bootstrap replicates) was based on the conserved region of the genome of 31 orthopoxviruses. Colored boxes highlight the 3 main clades of the vaccinia lineage. The red arrow points to HSPV, which groups within the HSPV/South American clade, together with the Brazilian IOC vaccine strain and the Brazilian field strains of vaccinia virus. The red circles indicate the position of different cowpox viruses, which are polyphyletic and group outside the vaccinia lineage. The red star indicates the position of variola virus. The scale bar indicates the number of substitutions per site. HSPV, horsepox virus; IOC, Instituto Oswaldo Cruz.

For the last decade, the use of NGS technologies has immensely contributed to our knowledge on the evolutionary relationship of several vaccinia virus strains (Fig 2). Genome sequencing of Dryvax and Instituto Oswaldo Cruz (IOC) clones (20th century smallpox vaccine strains used in the United States of America and Brazil, respectively) revealed genetic distances between clones (individual viruses isolated from a vaccine pool by plaque purification) that are similar to or even bigger than the distances between different vaccinia or variola strains [17, 18]. This suggests that 20th century smallpox vaccines may, in fact, represent a mixture of vaccinia strains, which substantiates historical reports. Intriguing patterns of recombinational events between clones may even have increased the genetic diversity of these strains [19].

Genomic analyses also provide evidence that horsepox virus is grouped within the South American clade of the vaccinia virus lineage, closely related to IOC and the feral vaccinia strain Cantagalo (Fig 2) [3, 18]. This is very intriguing, but the fact that there is only one extant specimen of horsepox (isolated from infected horses in Mongolia in 1976 and possibly extinct in Europe and Asia) does not provide much scope for conclusions [13, 20]. Several features of the horsepox genome, such as the presence of full-length genes that otherwise are truncated in all vaccinia strains, suggest that horsepox virus is probably closely related to an ancestor of the vaccinia lineage. However, the clustering within the vaccinia phylogenetic tree suggests that horsepox virus could be considered a vaccinia strain. Additionally, patches of horsepox sequences are found in some clones of Dryvax and IOC strains, suggesting recombination events and reinforcing the findings that both clades share a common ancestor or indicating previous copropagation [18, 19]. However, despite clustering within the vaccinia lineage, horsepox virus is distant from other vaccinia strains—its genome has striking features that are not found in any vaccinia virus. For example, horsepox virus has a 10.7-kb region at the left end and a 5.5-kb region at the right end of the genome that are missing in all known vaccinia strains [13].

Recently, the conclusion that there is a close relationship of horsepox and vaccinia gained support with the analysis of an old vaccine produced by the H. K. Mulford Company, Philadelphia, in 1902. The glycerinated content of a capillary was recovered, and genome sequencing revealed 99.7% similarity of the central core region with horsepox virus. However, the Mulford-1902 strain differs from horsepox in having the 2 large deletions in both genome ends that are typical of all vaccinia strains [14]. These findings provide the first scientific evidence that a horsepox-like virus was once used in the production of the smallpox vaccine, and because there is no record whatsoever of the circulation of horsepox virus in the Americas, the seed for the Mulford vaccine must originate in 19th century Europe.

### Conclusions and future perspectives

Many questions about the true nature of the smallpox vaccine produced before the mid-20th century remain unanswered. A significant advance resulted from the finding that horsepox virus was in fact used to produce at least one commercial smallpox vaccine, but we do not know if this was a one-time event or a widely utilized practice. Additionally, we still don’t know when and how vaccinia virus began to be used. And perhaps most astonishing, we don’t know if cowpox virus has ever been used as a smallpox vaccine because no existing vaccine contains this virus. Old drawings of children’s arms show vaccine lesions with a central black scar suggestive of cowpox infection, but secondary bacterial infections could have caused similar lesions. The main answers to our questions certainly reside in further studies of ancient smallpox vaccines. Genomic analyses of such samples will illuminate this intricate puzzle.
